# Overexpression of Teashirt Homolog 2 suppresses cell proliferation and predicts the favorable survival of Lung Adenocarcinoma

**DOI:** 10.7150/ijms.52109

**Published:** 2021-03-03

**Authors:** Shilei Zhao, Xin Guo, Ken-ichi Mizutani, Reimon Yamaguchi, Sohsuke Yamada, Chundong Gu, Hidetaka Uramoto

**Affiliations:** 1 Department of Thoracic Surgery, The First Affiliated Hospital of Dalian Medical University, Dalian, China.; 2 Department of Thoracic Surgery, Kanazawa Medical University, Ishikawa, Japan.; 3 Department of Pathology and Laboratory Medicine, Kanazawa Medical University, Ishikawa, Japan.; 4 Department of Dermatology, Kanazawa Medical University, Ishikawa, Japan.

**Keywords:** Teashirt homolog 2, proliferation, prognosis, lung adenocarcinoma

## Abstract

**Background:** Teashirt homolog 2 (TSHZ2) is essential for maintaining cellular homeostasis and regulating transcription on neoplasia development. However, the regulation of TSHZ2 in lung tumorigenesis and the underlying mechanisms remain unclear.

**Objective:** To evaluate the relationship between TSHZ2 expression in patients' tumor tissue and prognosis in lung adenocarcinoma.

**Methods:** TSHZ2 expression in different lung adenocarcinoma cell lines and human tissue were detected by Western blotting. The effect of TSHZ2 on cell proliferation, apoptosis and migration in lung adenocarcinoma cells was measured by CCK8, colony formation, flowcytometric analyses and wound-healing, respectively. TSHZ2 expression in different lung adenocarcinoma cell lines and human tissue from patients was detected using Western blotting and immunohistochemical staining. We also retrospectively analyzed 226 lung adenocarcinoma patients after surgical resection using immunohistochemical staining, and the association of TSHZ2 expression with the patient survival was evaluated.

**Results:** TSHZ2 was lowly expressed in certain lung adenocarcinoma cell lines (PC9 and B203L), but other cells showed a high expression. Low expression of TSHZ2 was also observed in most lung adenocarcinoma tissues compared with adjacent tissues. Furthermore, we found that the overexpression of TSHZ2 plasmids led to the dramatic inhibition of cell proliferation, colony formation ability, migration and apoptosis induction in PC9 lung adenocarcinoma cells. In contrast, no obvious effect was found when the TSHZ2 expression was down-regulated by si-TSHZ2. An elevated TSHZ2 expression was observed in 155(68.6%) tumor tissues samples of lung adenocarcinoma patients. Notably, the lung adenocarcinoma patients with a high TSHZ2 expression tended to have EGFR mutations less frequently and a preferable prognosis to those with a lower expression.

**Conclusion:** A high TSHZ2 expression inhabited cell proliferation and predicted a better prognosis of lung adenocarcinoma, possibly representing a useful therapeutic target for lung adenocarcinoma.

## Introduction

Lung cancer is the leading cause of cancer death worldwide 1,2. The incidence of lung adenocarcinoma, the predominant pathological lung neoplasm subtype, is increasing gradually 3. Surgery, radiotherapy and chemotherapy can reduce the risk of relapse for new tumors and death in patients with lung adenocarcinoma, but the survival rate remains low due to the potential for malignant proliferation, with the situation even worse in developing countries 4.

The tumorigenesis and development of lung adenocarcinoma is a process of complex and consecutive changes processes of gene mutation, protein modification, inactive enzyme activity and activation of signal transduction pathways that accumulate to cause and exacerbate this disease 5-9. This difficult situation narrows down therapeutic options and leads to unreliable treatment outcomes. Therefore, clarifying the molecular mechanisms involved and identifying novel candidate biomarkers are crucial for the effective treatment of lung adenocarcinoma.

Since the discovery of the first zinc finger proteins involved in 5SRNA regulation in 1982, a total of 30 different structural families containing zinc fingers have been found, existing widely in human, animals and plants 10, 11. Different species have different numbers of zinc finger motifs that form stable finger-like structures with zinc ions, and the vast majority typically function as interaction modules that bind to DNA, RNA, proteins, or other small, useful molecules. Variations in structure serve primarily to alter the binding specificity of a particular protein 12. In addition, zinc fingers have been found to be extremely useful in various therapeutic and research capacities 13, 14.

Teashirt homolog 2 (TSHZ2) is a member of the teashirt C2H2-type zinc-finger protein family containing five zinc-finger motifs, one homeobox motif and one coiled coil that can act as transcriptional repressors involved in developmental processes. At present, the TSHZ2 function is believed to be limited to Alzheimer's disease 15, congenital pelvi-ureteric junction obstruction 16 and craniosynostosis 17; other oncological functions have been rarely reported and may not even exist. In our previous studies 18, we performed a microarray expression analysis using a combination of tumor tissues and cell lines based on recurrence or no-recurrence cases, and detected the abnormal expression of TSHZ2 from microarray profiles. Furthermore, using support vector machine (SVM) models 19, we speculated the TSHZ2 may specific binding to EGFR promoters by virtue of zinc-finger motifs (see Fig. S1 for schematic diagram). These date provide clues that may guide follow-up research on the lung adenocarcinoma biological function and mechanism.

In the present study, we investigated the aberrant expression of TSHZ2 in lung adenocarcinoma cells. The up-regulation of TSHZ2 markedly inhibited the cell proliferative ability, migration and induced apoptosis *in vitro* but not in TSHZ2 down-regulated cells. We also examined the frequency and clinical significance of the TSHZ2 expression in a retrospective series of 226 patients who underwent lung adenocarcinoma resection and analyzed its impact on the patients' prognosis. Our results indicated that TSHZ2 exerts a tumor-suppressive effect on the progression of lung adenocarcinoma and may serve as a candidate therapeutic target in lung adenocarcinoma treatment.

## Material and methods

### Cell lines and cell culture

Immortalized human bronchial epithelial (HBE) cells and human lung adenocarcinoma (A549, H1573 and PC9) cells were obtained from the American Type Culture Collection (Manassas, VA, USA), and A110L and B203L cell lines were kindly gifted by Dr. Izumi H 20. All of the cells were cultured as monolayer in RPMI-1640 medium (HyClone; GE Healthcare Life Sciences, USA) or DMEM (HyClone; GE Healthcare Life Sciences, USA) supplemented with 10% fetal bovine serum (FBS; Gibco, Waltham, MA, USA), and maintained in an environment with a humidified incubator under 5% CO2 at 37 °C.

### Western blot

Collect cells at the cell culture time or tumor tissues from the four patients with lung adenocarcinoma in a microcentrifuge tube. Total protein was collected and quantified using a BCA protein assay kit (Thermo Fisher Scientific, Waltham, MA, USA) as in a previous article 21. Protein lysates (10-20 g) were then separated by 8-12% sodium dodecyl sulfate-polyacrylamide gel electrophoresis (SDS-PAGE) and transferred to a polyvinylidene difluoride (PVDF) membrane. The samples were incubated with the specific antibodies (appropriate concentration referred to specification) and the protein band was detected with an enhanced chemiluminescence system.

### Plasmid constructs and transfection of human lung adenocarcinoma cells

pDsRed-monomer-C1-tagged human TSHZ2 was purchased from Clontech and kindly provided by Kasai K from Aichi Medical University School of Medicine, Nagakute 22. PC9 cells were transfected with pDsRed-monomer-C1-TSHZ2 or pDsRed-monomer-C1-vector in 6-well plates (200,000 cells per well). The siRNAs targeting TSHZ2 (siRNA1: 5′-GAGGCCUGCAAGUCCCAGAUCUUAA′-3; 5′-UUAAGAUCUGGGACUUGCAGGCCUC-3′. siRNA2: 5′-CCACAAGAGCGUAUGCAAAUCUCUA-3′, 5′-UAGAGAUUUGCAUACGCUCUUGUGG-3′. siRNA3: 5′-CAUUUGUGAGCAAACAUGCGGUAAA-3′, 5′-UUUACCGCAUGUUUGCUCACAAAUG-3′) and negative control siRNA were purchased from Invitrogen (Thermo Fisher Scientific, Waltham, MA, USA). A549 cells were transfected with siRNA duplexes (1-2 μg) in 6-well plates (200,000 cells per well). In addition, we found that siRNA1 had the best down-regulating effect on TSHZ2 at the preliminary pre-experiment, so we used it in subsequent experiments. At 72 h after pDsRed-monomer-C1-TSHZ2 or siRNA-TSHZ2 treatment, protein or cells were gathered for testing and verification by requisite assays.

### Cell viability assay

A total of 100 μl of transfected cells suspension was prepared and seeded in 96-well plates at a standard of 2,000 cells/well. CCK-8 reagent (10 μl; Dojindo Molecular Technologies, Inc. Japan.) was added to each well, and then the samples were incubated for 2 h at 37 °C. The cell viability was detected at 24, 48 and 72 h at 450 nm excitation using a microplate spectrophotometer.

### Clone formation assay

A total of 1,000 transfected cells were seeded into 12-well plates (200 cells/well, 3 paralleled wells). After 14 days, the samples were fixed with methanol and then stained with 0.1% crystal violet. An effective clone containing more than 50 cells were manually counted using a digital camera.

### Apoptosis assay

Following transfection, programmed cell death of samples was evaluated based on annexin V (AV) and propidium iodide (PI) according to the manufacturer's instructions (Thermo Fisher Scientific, Waltham, MA, USA). In brief, the cells (1×10^6^ cells/ml) were collected and then resuspend in 100μl 1 X Annexin V binding buffer. AV (5 μL) and 1 μL 100 μg/mL PI working solution were then added to 100 μL of cell suspension. After 15 min incubation, we added 400 μL 1X annexin-binding buffer and mixed again. Finally, we conducted flow cytometry (Becton, Dickinson and Company, USA) and performed analyses using the Flowing program (Perttu Terho, the Turku Centre for Biotechnology).

### Wound healing

Cells in a six-well plate were cultured and grown to full confluence. The plate was then scratched with the tip of a 200 µL pipette in order to create a cell-free region. Cells were cultured in 2% FBS medium. After 24 and 48 h, the width of the scarification was observed under a light microscope. The migration rate (MR) was calculated as follows: [initial gap (0h) - terminal gap (48h) / initial gap (0h)] ×100%.

### Immunohistochemical (IHC) staining

All resected specimens were obtained from primary lesions, fixed with formalin, and embedded the sections were then briefly incubated with xylene, rehydrated with graded ethanol solutions, incubated with methyl alcohol containing 3% hydrogen peroxide and immersed in a citrate buffer for antigen retrieval as previously described. IHC staining of TSHZ2 was performed in primary lesions and normal adjacent tissues according to the manufacturer's instructions for the streptavidin-peroxidase staining kit. Antibodies of polyclonal TSHZ2 antibody were diluted 1:200 in phosphate-buffered saline (PBS) containing 2% goat bovine serum.

Immunostaining was evaluated by two pulmonary pathologists (Dr. Mizutani and Dr. Yamada) using a blind protocol design. For each specimen, the total score of the expression intensity of TSHZ2 (negative staining: 0 points; weak staining: 1 points; moderate staining: 2 points; and strong staining: 3 points) multiplied by the stained cell numbers (≤25% of cells positive: 1 points; 26%-50% of t cells positive: 2 points; 51%-75% of cells positive: 3 points; >75% of cells positive: 4 points) was estimated. Samples with the a score ≥6 points, were defined as showing high expression, all other point values indicated a low expression. TSHZ2 positive and negative controls were referenced to breast cancer and liver cancer, respectively on the proteinatlas website (http://www.proteinatlas.org).

### Patients and follow-up

A total of 226 consecutive patients(median age: 68.7 years old; range: 37-83 years old) who underwent radical surgery of the primary tumor and systematic lymph node dissection or sampling at the Department of Thoracic Surgery of Kanazawa Medical University from January 2005 to December 2015 were included in this study, and 85 of them had information on EGFR mutations. In addition, all of the patients were confirmed to have lung adenocarcinoma by postoperative pathology (referring to the eighth edition tumor node metastasis [TNM] classification for NSCLC), and all had complete clinical data available with none receiving chemotherapy or radiotherapy prior to the operation. The hospital institutional review board of Kanazawa Medical University approved the protocol (I159).

All enrolled patients were followed up according to our previous study protocol 21, 23. The postoperative follow-up time points were every three months within the first year and every six months thereafter. During the follow-up, a physical examination, chest radiography, analysis of blood chemistry and carcinoembryonic antigen assay were performed. If any symptoms or signs of recurrence appeared in these examinations, further evaluations to detect the recurrent site were carried out. Follow-up time was terminated in May 2017 (median follow-up: 46 months).

### Statistical analyses

Student's t-test or an analysis of variance (ANOVA) was used to compare the values of the test and control samples *in vitro* and *in vivo*. The associations between the TSHZ2 expression and categorical variables were compared by Pearson's chi-squared test. Survival curves were plotted according to the Kaplan-Meier method, and differences between the curves were analyzed by the log-rank test. The Cox proportional hazards model was applied to the multivariate survival analysis. Differences were considered to be statistically significant for p-values of <0.05. Data were analyzed using the SPSS 22 software program (IBM Corporation, Armonk, NY, USA).

## Results

### The expression of TSHZ2 in lung adenocarcinoma cell lines and tissues

**Figure 1:**
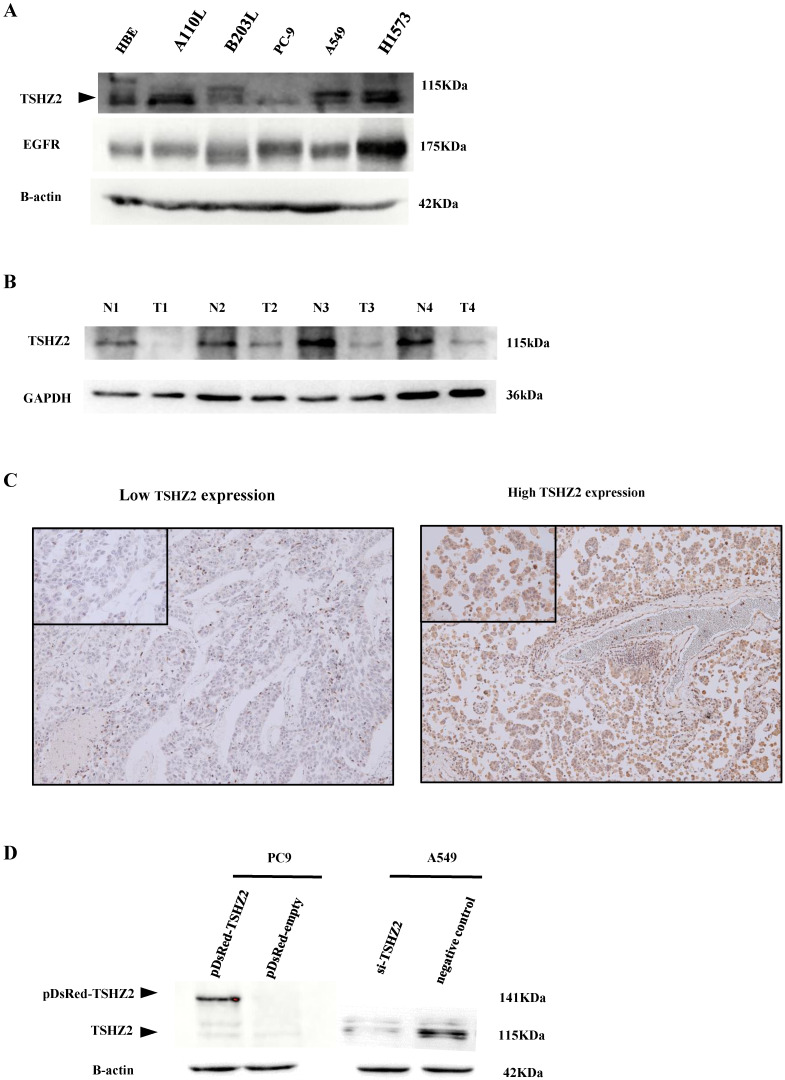
The expression of TSHZ2 or EGFR in human lung adenocarcinoma cells and tissues. **A.** An analysis of the total protein extracted from different human lung adenocarcinoma lines by Western blotting. **B.** The protein levels of TSHZ2 detected in tumor and adjacent tissues from the four patients with lung adenocarcinoma. **C.** The representative images of immunohistochemical analyses of TSHZ2 in human lung adenocarcinoma tissues (original magnification: ×100; inset ×400). **D.** An analyze of the TSHZ2 expression at 72 h after transfecting pDsRed-monomer-C1-TSHZ2 (pDsRed-TSHZ2)/pDsRed-monomer-C1-vector (pDsRed-empty) or siRNA-TSHZ2/siRNA-negative control to PC9 or A549 cells by Western blotting. Calculations were repeated three times.

The expression of TSHZ2 was first detected in lung adenocarcinoma cells and tissues. A Western blot analysis indicated that TSHZ2 was expressed in various lung adenocarcinoma cell lines (Fig. 1A). A110L, H1573 and A549 cells had obvious TSHZ2 expression, but PC9 and B203L cells had little expression of TSHZ2; the expression in immortalized HBE cells fell in between these two extremes. Of note, the TSHZ2 expression was negatively correlated with the EGFR expression in most adenocarcinoma cells except for H1573 adenocarcinoma cells. Furthermore, bioinformatics predicted the relationship between TSHZ2 and EGFR (see Fig. S1 for schematic diagram).

Low expression of TSHZ2 was observed from the four patients in lung adenocarcinoma tissues compared with adjacent tissues (Fig. 1B). We also analyzed the expression of TSHZ2 in 226 patients who had undergone complete surgical resection of lung adenocarcinoma by IHC staining. Among these patients, 155 (68.6%) had a high TSHZ2 expression predominantly located in the nucleus and a small portion in the cytoplasm. Representative staining images are shown in Fig. 1C.

### Induction of the TSHZ2 expression in different adenocarcinoma cell lines

Due to the differences in the TSHZ2 expression among adenocarcinoma cell lines, we subsequently transfected pDsRed-monomer-C1-TSHZ2 or siRNA-TSHZ2 into PC9 or A549 cells, respectively, and verified the results by Western blotting. We found an increased TSHZ2 expression in pDsRed-monomer-C1-TSHZ2 treated PC9 cells, but its molecular weight (141kDa) is higher due to the fusion of the red fluorescent protein. In contrast, a reduced TSHZ2 (molecular weight 115kDa) was detected in siRNA-TSHZ2 treated A549 cells (Fig. 1D).

### Regulation of the proliferation, apoptosis and migration by TSHZ2 in lung adenocarcinoma *in vitro*

**Figure 2:**
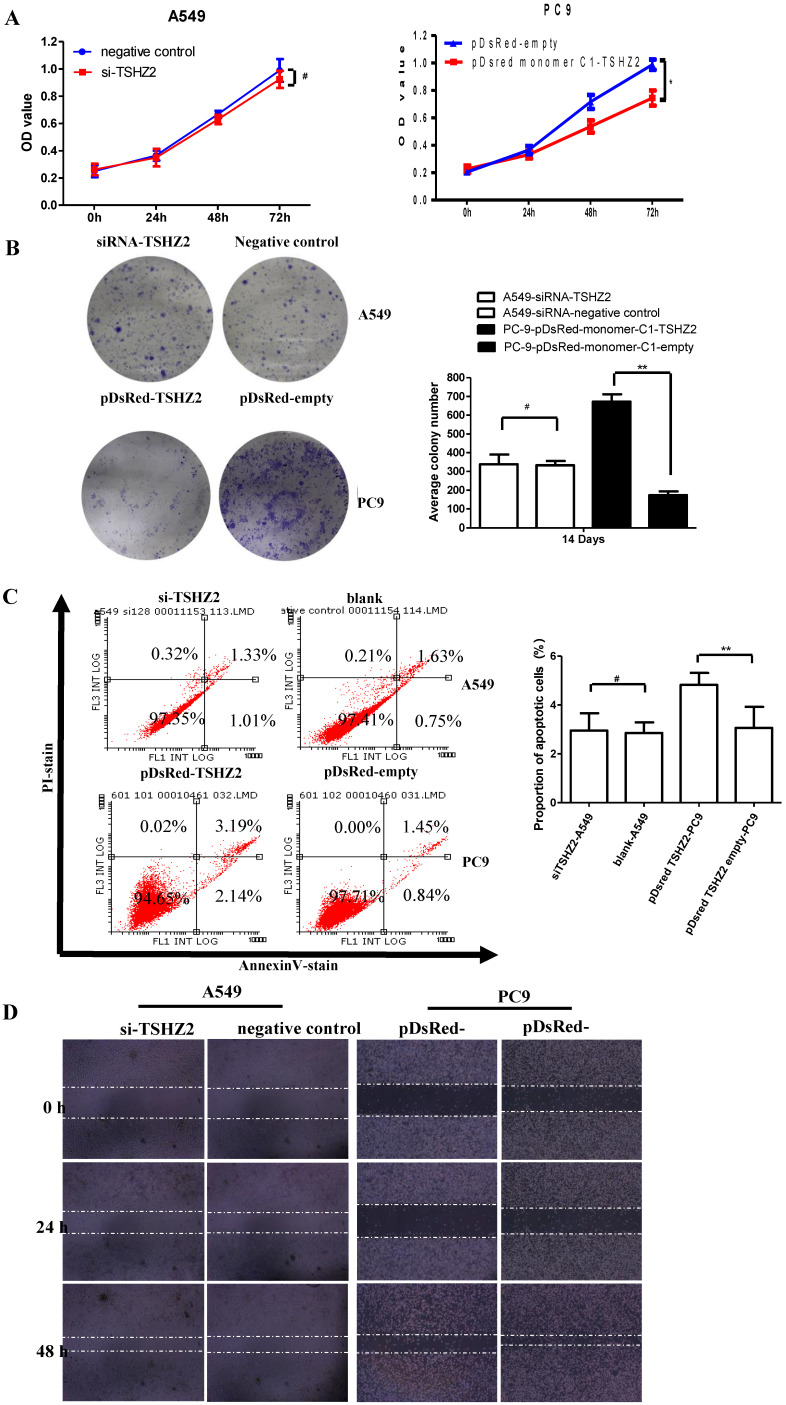
Effects of the TSHZ2 expression on the proliferation, apoptosis and migration of A549 and PC9 cells after transfection with pDsred monomer C1-empty/pDsred monomer C1-TSHZ2 or siRNA-TSHZ2 /siRNA-negative control. **A.** The cell proliferation of siRNA treated A549 and pDsRed-monomer-C1 treated PC9 cells at 24, 48 and 72h was detected using the CCK-8 assay. **B.** Colony formation assay of siRNA treated A549 and pDsRed-monomer-C1-treated PC9 cells at 14 days. **C.** siRNA- treated A549 and pDsRed-monomer-C1-treated PC9 cells at 48h; apoptosis was determined by a FACS analysis. **D.** Cell migration was analyzed by a wound-healing assay. siRNA treated A549 and pDsRed-monomer-C1 treated PC9 cells were seeded in six-well plates and grown to full confluence. Experiments were repeated 3 times and presented as the mean±SD (*p<0.05, **p≤0.001 and^#^ *p>*0.05).

We next examined the effect of TSHZ2 on the cell viability by a CCK8 and clone formation assay. As shown in Fig. 2A, the overexpression of TSHZ2 in PC9 cells significantly suppressed the cell viability compared to the cells treated with pDsred monomer C1-empty. Consistent with the results from the inhibition of clone formation, the pDsred monomer C1-TSHZ2-treated cells formed nearly 100 clones after 14 days' growth, which was less than in cells treated with other agent (Fig. 2B).

We also determined the effect of TSHZ2 on apoptosis by a flowcytometric analysis (Fig. 2C). The results showed that, at 72 h after transfection of pDsred monomer C1-TSHZ2, the number of PC9 apoptotic cells was greater than in the control groups. As shown in ​Fig. 2D, the wound-healing assay also revealed the inhibition effect of the pDsred monomer C1-TSHZ2 on tumor cell mobility in PC9 cells. However, siRNA-TSHZ2 treated A549 cells didn't drive theoretical function containing accelerated proliferation or migration and apoptosis resistance.

Similar results were obtained with siRNA treated H1573 cells (see Fig. S2). These results suggest that an elevated TSHZ2 expresssion restricts cell proliferation and migration and accelerates apoptosis in lung adenocarcinoma cells.

### Relationship of the TSHZ2 expression with clinicopathologic factors in lung adenocarcinoma and the survival of patients

The clinicopathologic characteristics of the patients were summarized in Table 1. The expression of TSHZ2 was significantly associated with the smoking index (*p=*0.008) and differentiation (*p=*0.005), but not sex, age, lymphatic vessel invasion, vascular invasion, TNM stage, operation method or Ki67 level. Furthermore, TSHZ2 had a negative correlation with EGFR mutations (*p=*0.035) as shown in Table S3, but no differences in expression were noted among EGFR mutation types (*p=*0.591, Table S4).

**Figure 3:**
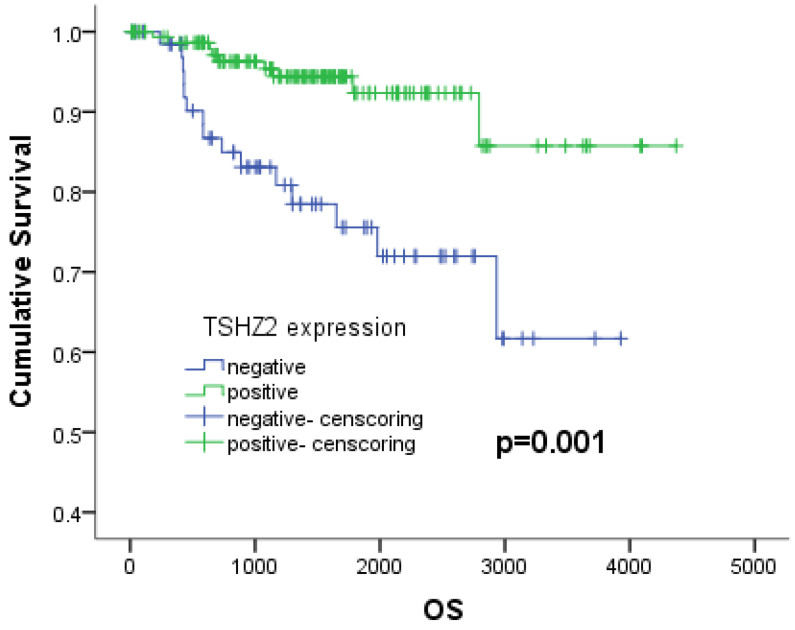
The 5-years survival depending on the expression of TSHZ2. The 5-year survival rates with negative and positive expression were 75.6% and 92.3%, respectively (*p=*0.001).

We next analyzed the relationship between the clinicopathologic factors and prognosis in 226 patients with lung adenocarcinoma. A Kaplan-Meier analysis showed that patients with a high smoking index (*p<*0.001), no- cVATS operation (*p=*0.039), high TNM stage (*p<*0.001), large tumor size (*p=*0.001), poor differentiation (*p<*0.001), vascular invasion (*p=*0.001), negative TSHZ2 expression (*p=*0.001) or a high Ki67 expression (*p=*0.001) had a poorer survival outcome than others, according to a log-rank test (Table 2). A multivariate Cox proportional hazards model analysis of the risk factors showed that a low TNM stage (stage I: odds ratio [OR]: 0.144, 95% confidence interval [CI]: 0.049-0.424, *p<*0.001; stage II: OR: 0.315, 95% CI: 0.110-0.905, *p<*0.001) and positive TSHZ2 expression (OR=0.382, 95%CI: 0.160-0.913, *p=*0.030) were independent prognostic factors for lung adenocarcinoma (Table 3). The 5-year overall survival with a positive TSHZ2 expression was 92.3%, which was significantly longer than that with a negative expression (75.6%) using the Kaplan Meier method (Fig. 3).

## Discussion

Regarding the role of the Teashirt C2H2-type zinc-finger protein family in the regulation of various biological functions, including cellular differentiation, DNA-binding and signal transmission, the function and molecular mechanisms of each member in this family deserve to be more thoroughly investigated in human diseases, especially cancer initiation and progression 24. However, most studies thus far have focused on TSHZ3 25, with only sporadic reports available concerning TSHZ1 and TSHZ2 in non-neoplastic diseases. In our previous studies 18, we performed a microarray expression analysis using a combination of tumor tissues and cell lines based on recurrence or no-recurrence cases; we found that TSHZ2 was up-regulated more than four-fold in one patient with high recurrence and metastasis. However, its role as a candidate biomarker needs to be verified in large-scale study due to the selection bias associated with including only one case in the previous study. A further study of its function and molecular mechanism will expand our understanding of TSHZ2 and provide clues concerning its function in other tumors.

We reported for the first time the TSHZ2 expression in lung adenocarcinoma cell lines and large-scale samples, finding that the overexpression of TSHZ2 markedly inhibited lung adenocarcinoma cell growth and migration and induced tumor cell apoptosis *in vitro*. Furthermore, the elevated expression of TSHZ2 exerted as a tumor-inhibiting function, thereby increasing the lung adenocarcinoma survival. However, inhibiting the expression of TSHZ2 did not enhance the neoplastic malignancy in A549 or H1573 cells, we thought that because the tumorous malignant activity was already at a plateau. Overcoming this bottleneck requires long-term lasting endogenous or exogenous stimulation 26, siRNA obviously couldn't accomplish it in the short term.

The treatment effect of tyrosine kinase inhibitors (TKIs) has been verified in non-small cell lung cancer (NSCLC) patients with EGFR mutations 27, 28. With the advent of TKI drugs, postoperative quality of life and prognosis of patients with lung adenocarcinoma have been significantly improved. However, EGFR wild-type patients enjoy little benefit from these agents. We previously found that TSHZ2 had a negative correlation with EGFR mutations, with EGFR wild-type patients with lung adenocarcinoma showing an expression rate of 32.4%. Therefore, increasing the TSHZ2 expression in EGFR wild-type patients with lung adenocarcinoma might be a viable alternative treatment option. We also found that that the TSHZ2 expression was negatively correlated with the EGFR expression in most lung adenocarcinoma cell lines. Using the SVM models 19 we predicted the DNA recognition of TSHZ2 and identified four possible binding regions on the EGFR promoter. TSHZ2 may therefore act as a transcriptional repressor to suppress the EGFR expression and restrict the activation of downstream signaling pathways 29, 30. The specific molecular mechanisms will be elucidated in our follow-up studies.

Through our retrospective analysis of 226 cases of lung adenocarcinoma, a high expression of TSHZ2 was found to be significantly associated with the smoking index (*p=*0.008) and differentiation (*p=*0.005) but no other clinicopathological factors. These two risk factors often indicated a poor prognosis. Furthermore, we found that a low TSHZ2 expression was an independent risk factor affecting the prognosis in along with the TNM stage and smoking index in univariate and multivariate analyses. These results conflict with our previous findings in recurrence cases according to an RNA-microarray expression analysis. This discrepancy may have been due to RNA-transcription and protein-translation differences in the TSHZ2 gene and the limited number of cases evaluated. We then used the bioinformatic analyses software (http://gepia2.cancer-pku.cn/#index) to further prove the validity of our data. The RNA expression of TSHZ2 in tumor was significantly lower than normal tissue and the overall survival of patients with the higher TSHZ2 expression was lower (Fig. ​4). In addition, Yamamoto et al 24 showed that TSHZ2 was a tumor suppressor gene in breast and prostate cancers, which was similar to our actual results.

## Conclusion

In summary, we provided functional evidence that a high TSHZ2 expression suppresses lung adenocarcinoma progression *in vitro*. Furthermore, the negative expression of TSHZ2 function as an independent risk factor for the overall survival of patients with lung adenocarcinoma. Taken together, these findings suggest that a high TSHZ2 expression may serve as a biomarker predicting a favorable prognosis and may be as a potential therapeutic target for patients suffering from lung adenocarcinoma.

## Supplementary Material

Supplementary figures and tables.

## Figures and Tables

**Figure 4:**
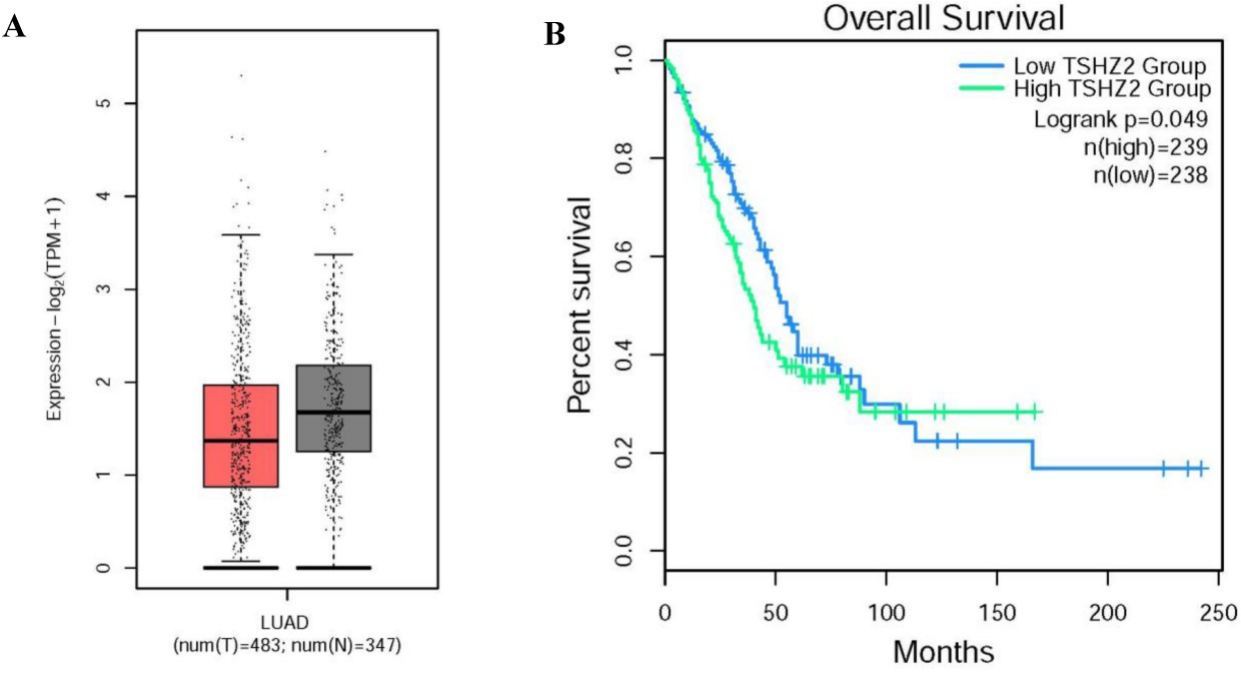
The different expression and overall survival of TSHZ2 in human lung adenocarcinoma used the TCGA normal and GTEx data (http://gepia2.cancer-pku.cn/#index). **A.** The TSHZ2 RNA expression in human lung adenocarcinoma (n=483) and normal tissue (n=347). **B.** The overall survival of patients with the low or high TSHZ2.

**Table 1:** Relations between the expression of TSHZ2 and clinicopathologic characteristics in 226 patients with lung adenocarcinoma

Factor	TSHZ2 expression		*p*-value
	Negative (%)	Positive (%)	
**Gender**			0.191
Male	41 (35.3%)	75 (64.7%)	
Female	30 (27.3%)	80 (72.7%)	
**Age**			0.316
≤68.7	32 (28.3%)	81 (71.7%)	
>68.7	39 (34.5%)	74 (65.5%)	
**Smoking index**			**0.008**
<400	34 (24.8%)	103 (75.2%)	
≥400	37 (41.6%)	52 (58.4%)	
**Location (lung lobe)**			0.460
Right-up	24 (30.8%)	54 (69.2%)	
Right-mid	5 (22.7%)	17 (77.3%)	
Right-down	18 (40.0%)	27 (60.0%)	
Left-up	12 (25.0%)	36 (75.0%)	
Left-down	12 (36.4%)	21 (63.6%)	
**Operation method**			0.443
cVATS	0 (00.0%)	2 (100.0%)	
hybrid	63 (30.9%)	141 (69.1%)	
thoracotomy	8 (40.0%)	12 (60.0%)	
**TNM stage**			0.051
I	51 (28.9%)	128 (71.1%)	
II	14 (51.9%)	13 (48.1%)	
III	6 (30.0%)	14 (70.0%)	
**Tmax**			0.521
≤1 cm	1 (14.3%)	6 (85.7%)	
>1 cm, ≤2 cm	29 (35.4%)	53 (64.6%)	
>2 cm, ≤3 cm	23 (32.9%)	47 (67.1%)	
>3 cm	18 (26.9%)	49 (73.1%)	
**Differentiation**			**0.005**
High	23 (21.1%)	86 (78.9%)	
Mid	38 (40.4%)	56 (59.6%)	
Poor	10 (43.5%)	13 (56.5%)	
**Lymphatic vessel invasion**			0.550
Without	41 (29.9%)	96 (70.1%)	
With	39 (39.8%)	59 (60.2%)	
**Vascular invasion**			0.712
Without	44 (30.6%)	100 (69.4%)	
With	27 (32.9%)	55 (67.1%)	
**Ki67**			0.513
≤10%	37 (29.6%)	88 (70.4%)	
>10%	34 (33.7%)	67 (66.3%)	

Notes: TNM: tumor, node, metastases; Tmax: maximum diameter of tumor; Ki67: proliferation marker protein Ki-67; TSHZ2: teashirt homolog 2; cVATS: complete video-assisted thoracic surgery; hybrid: video-assisted thoracic surgery with thoracotomy.

**Table 2:** Univariate analysis of 5-year OS on different clinicopathological factors using Kaplan-Meier method

Risk Factor	5-OS%	Log rank (*p*-value)
**Gender**		**0.002**
Male	78.9	
Female	95.3	
**Age**		0.103
≤68.7	90.6	
>68.7	83.0	
**Smoking index**		**<0.001**
<400	94.5	
≥400	73.9	
**Location (lung lobe)**		0.286
Right-up	89.2	
Right-mid	95.0	
Right-down	84.5	
Left-up	87.7	
Left-down	81.3	
**Operation method**		**0.039**
cVATS	100.0	
Hybrid	88.5	
Thoracotomy	72.4	
**TNM stage**		**<0.001**
I	94.1	
II	70.2	
III	51.8	
**Tmax**		**0.001**
≤1 cm	100.0	
>1 cm, ≤2 cm	94.0	
>2 cm, ≤3 cm	94.4	
>3 cm	73.1	
**Differentiation**		**<0.001**
High	97.6	
Mid	78.1	
Poor	73.2	
**TSHZ2**		**0.001**
Negative	75.6	
Positive	92.3	
**Lymphatic vessel invasion**		0.365
Without	88.6	
With	84.6	
**Vascular invasion**		**0.001**
Without	92.5	
With	75.9	
**Ki67**		**0.001**
≤10%	92.5	
>10%	80.9	

Notes: TNM: tumor, node, metastases; Tmax: maximum diameter of tumor; Ki67: proliferation marker protein Ki-67; TSHZ2: teashirt homolog 2; cVATS: complete video-assisted thoracic surgery; hybrid: video-assisted thoracic surgery with thoracotomy; 5-OS%: 5-year overall survival.

**Table 3:** Multivariate analysis of overall survival by Cox-regression

Factor	OR	95%CI	*p*-value
**Gender**			
Male	1		
Female	0.620	0.168-2.287	0.473
**Smoking index**			
<400	1		
≥400	4.535	1.726-11.916	**0.002**
**Operation method**			
Thoracotomy	1		
cVATS& hybrid	1.000	0.335-2.982	1.000
**TNM stage**			
I	0.144	0.049-0.424	**<0.001**
II	0.315	0.110-0.905	**0.032**
III	1		
**Tmax**			
≤3 cm	1		
>3 cm	2.307	0.872-6.106	0.092
**Differentiation**			
High	0.121	0.012-1.209	0.072
Mid	0.956	0.319-2.943	0.956
Poor	1		
**TSHZ2**			
Negative	1		
Positive	0.382	0.160-0.913	**0.030**
**Vascular invasion**			
Without	1		
With	1.155	0.426-3.131	0.777
**Ki67**			
≤10%	1		
>10%	1.559	0.503-4.831	0.441

Notes: TNM: tumor, node, metastases; Tmax: maximum diameter of tumor; Ki67: proliferation marker protein Ki-67; TSHZ2: teashirt homolog 2; cVATS: complete video-assisted thoracic surgery; hybrid: video-assisted thoracic surgery with thoracotomy; 5-OS%: 5-year overall survival.
