# Supplementary material for: Overexpression of Teashirt Homolog 2 suppresses cell proliferation and predicts the favorable survival of Lung Adenocarcinoma
Source: Int J Med Sci. 2021 Mar 3;18(9):1980–9. doi: 10.7150/ijms.52109 (PMC8040402; doi:10.7150/ijms.52109)
Supplement: Supplementary file 1 — Supplementary figures and tables. [file ijmsv18p1980s1.pdf]

S1 Fig

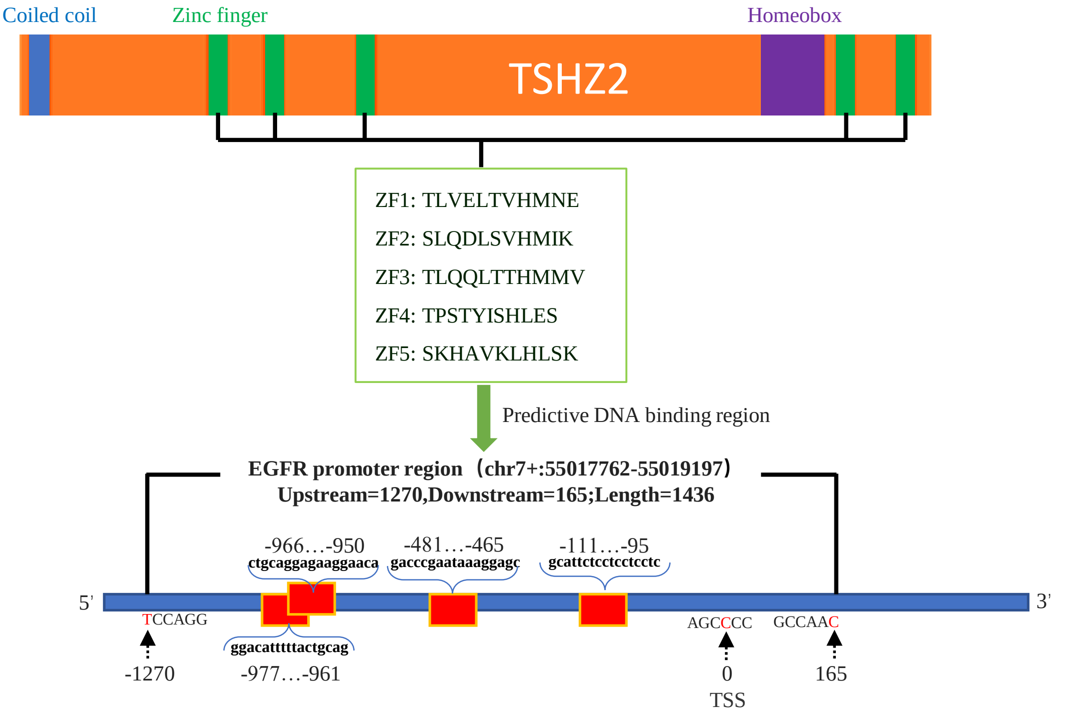
Coiled coil
Zinc finger
Homeobox
TSHZ2
ZF1: TLVELTVHMNE
ZF2: SLQDLSVHMIK
ZF3: TLQQLTTHMMV
ZF4: TPSTYISHLES
ZF5: SKHAVKLHLSK
Predictive DNA binding region
EGFR promoter region（chr7+:55017762-55019197）
Upstream=1270,Downstream=165;Length=1436
-966…-950
ctgcaggagaaggaaca
-481…-465
gacccgaataaaggagc
-111…-95
gcattctcctcctcctc
5'
3'
TCCAGG
ggacatttttactgcag
AGCCCC
GCCAAC
-1270
-977…-961
0
TSS
165

S2 Fig

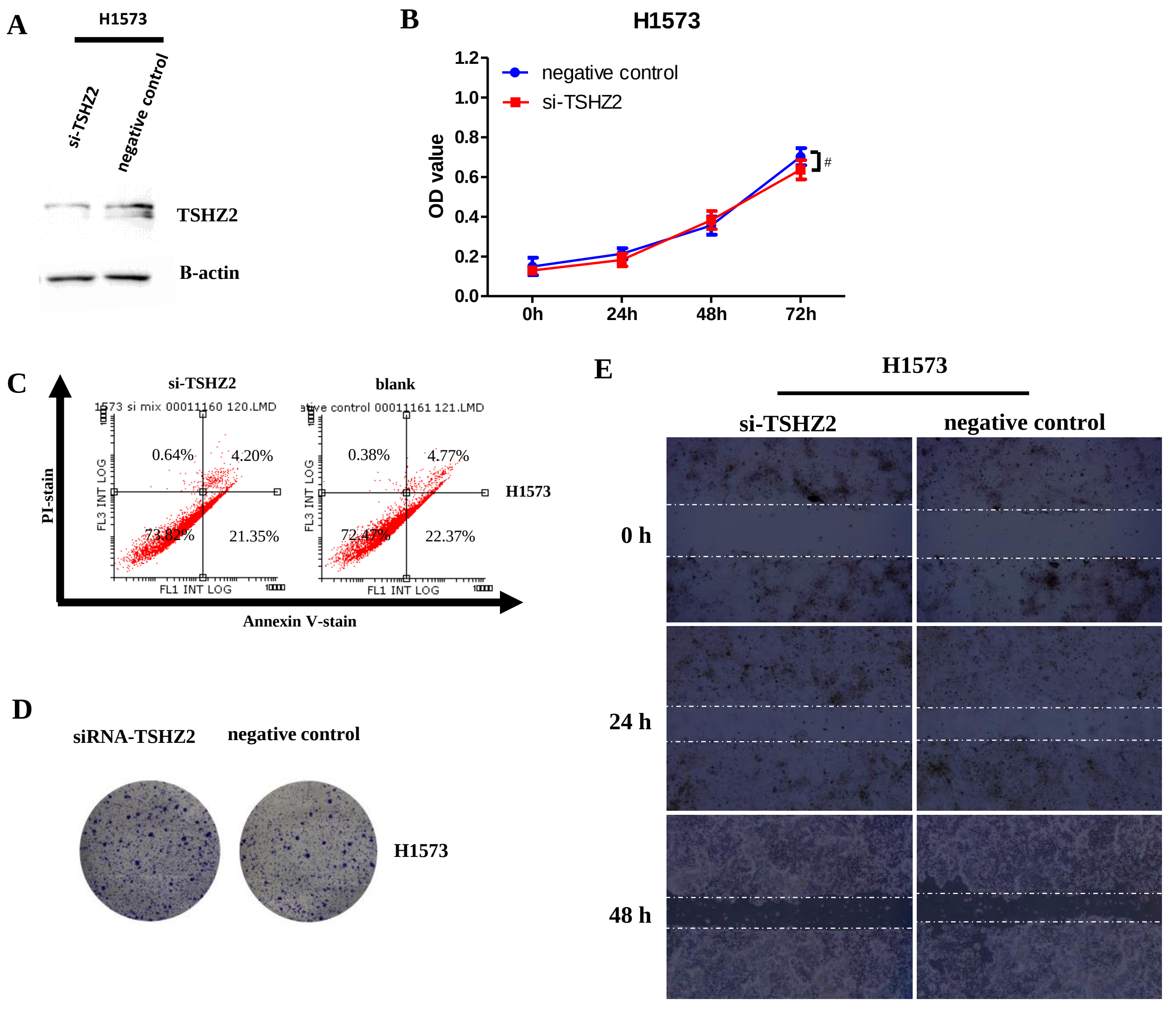
A
H1573
si-TSHZ2
negative control
TSHZ2
B-actin
B
H1573
negative control
si-TSHZ2
OD value
1.2
1.0
0.8
0.6
0.4
0.2
0.0
0h
24h
48h
72h
#
C
si-TSHZ2
blank
PI-stain
0.64%
4.20%
73.82%
21.35%
0.38%
4.77%
72.47%
22.37%
FL3 INT LOG
FL1 INT LOG
H1573
Annexin V-stain
D
siRNA-TSHZ2
negative control
H1573
E
H1573
si-TSHZ2
negative control
0 h
24 h
48 h

S1 Fig. Schematic graph of TSHZ2 binding to the EGFR promoter. Five zinc finger structure areas (green) were derived from TSHZ2. Four TSHZ2 specific binding domain (red) were speculated on EGFR promoter regions. The information of EGFR promoter was obtained from www.genecopoeia.com.

S2 Fig. Effects of the down-regulation of the TSHZ2 expression on the proliferation , apoptosis and migration of H1573 cells after transfection with siRNA-TSHZ2 or siRNA-negative control. A. An analysis of the TSZH2 expression from the total protein of siRNA-treated H1573 cells by Western blot. B. The cell proliferation at 24 and 48h was detected using the CCK-8 assay. C. The apoptosis was determined by a FACS analysis. D. A colony formation assay was performed at 14 days. E. Cell migration was analyzed by a wound-healing assay. H1573 cells were seeded into six-well plates and grown to full confluence. Experiments were repeated 3 times and presented as the mean±SD (*p<0.05, **p=<0.001 and# p>0.005).

S3 Table. The correlation between EGFR mutations and TSHZ2 protein expression in 153 lung adenocarcinoma specimens.

Factor	TSHZ2		p-value
	Negative (%)	Positive (%)	
**EGFR**			
**Wild**	22 (32.4%)	46 (67.6%)	0.035
**Mutant**	15 (17.6%)	70 (82.4%)	

Notes: EGFR: epidermal growth factor receptor; TSHZ2: teashirt homolog 2.

S4 Table. The correlation between different types of EGFR mutations and TSHZ2 expression.

EGFR-mutation type	N=85	TSHZ2		p-value
		Negative (%)	Positive (%)	
**Insert in19 exon**	34	8(23.5%)	26(76.5%)	0.591
**Deletion in19 exon**	1	0(0.0%)	1(100.0%)	
**Point mutation in 21 exon**	3	0(0.0%)	3(100.0%)	
**Other exon mutation**	47	7(16.3%)	40(83.7%)	

Notes: EGFR: epidermal growth factor receptor; TSHZ2: teashirt homolog 2.
